# Ceftriaxone resistant *Salmonella enterica* serovar Paratyphi A identified in a case of enteric fever: first case report from Pakistan

**DOI:** 10.1186/s12879-023-08152-9

**Published:** 2023-04-26

**Authors:** Seema Irfan, Zahra Hasan, Farah Qamar, Najia Ghanchi, Javaria Ashraf, Akbar Kanji, Safina Abdul Razzak, David Greig, Satheesh Nair, Rumina Hasan

**Affiliations:** 1 Department of Pathology and Laboratory Medicine, The Aga Khan University, Stadium Road, P.O Box3500, Karachi, 74800 Pakistan; 2 Department of Pathology and Laboratory Medicine, The Aga Khan University, Karachi, Pakistan; 3 Department of Pediatrics and Child Health, The Aga Khan University, Karachi, Pakistan; 4 Gastrointestinal Bacteria Reference Unit, UK Health Security Agency, London, UK; 5 GASTROINTESTINAL PATHOGENS UNIT Gastrointestinal Bacteria Reference Unit National Infection Service, Public Health England, London, UK; 6 Department of Pathology and Laboratory Medicine, The Aga Khan University, Karachi, Pakistan; 7 Faculty of Public Health and Policy, London School of Hygiene and Tropical Medicine, London, UK

**Keywords:** Ceftriaxone resistant *Salmonella* Paratyphi A, Case report, *Bla*_*CTX-M-15*_ in *S.* Paratyphi A, ESBL *S.* Para A, *S.* Para A from Pakistan, Enteric fever in Pakistan, Antimicrobial resistance (AMR) in Pakistan, Drug resistance in *Salmonella*

## Abstract

**Background:**

Enteric fever is an acute systemic infectious disease associated with substantial morbidity and mortality in low- and middle-income countries (LMIC), with a global burden of 14.3 million cases. Cases of enteric fever or paratyphoid fever, caused by *Salmonella enterica* serovar Paratyphi A (*S*. Para A) have been found to rise in many endemic and non-endemic countries. Drug resistance is relatively uncommon in* S*. Para A. Here we report a case of paratyphoid fever caused by ceftriaxone resistant *S*. Para A from Pakistan.

**Case presentation:**

A 29-year-old female presented with a history of fever, headache, and shivering. Her blood culture revealed a *S*. Para A isolate (S7), which was resistant to ceftriaxone, cefixime, ampicillin and ciprofloxacin. She was prescribed oral Azithromycin for 10 days, which resulted in resolution of her symptoms.

Two other isolates of *S*. Para A (S1 and S4), resistant to fluoroquinolone were also selected for comparison. DST and whole genome sequencing was performed for all three isolates. Sequence analysis was performed for identification of drug resistance and phylogeny. Whole Genome Sequencing (WGS) of S7 revealed the presence of plasmids, IncX4 and IncFIB(K). blaCTX-M-15 and qnrS1 genes were found on IncFIB(K). The *gyr*A S83F mutation conferring fluoroquinolone resistance was also found present. Multi-locus sequence typing (MLST) showed the S7 isolate to belong to ST129. S1 and S4 had the *gyr*A S83Y and S83F mutations respectively.

**Conclusions:**

We highlight the occurrence of plasmid-mediated ceftriaxone resistant strain of* S*. Para A. This is of significance as ceftriaxone is commonly used to treat paratyphoid fever and resistance in *S*. Para A is not known. Continuous epidemiological surveillance is required to monitor the transmission and spread of antimicrobial resistance (AMR) among Typhoidal Salmonellae. This will guide treatment options and preventive measures including the need for vaccination against *S*. Para A in the region.

## Background

Enteric fever (Typhoid and Paratyphoid fever) is an acute systemic infectious disease. It is associated with a global burden of 14·3 million cases, causing substantial morbidity and mortality (*1*). Since in low- and middle-income countries (LMIC), a rising trend of antimicrobial resistance is seen in *Salmonella* species, WHO has ranked it as a high priority organism requiring research and newer antibiotic development (https://www.who.int/publications/i/item/WHO-EMP-IAU-2017.12). *Salmonella enterica* serovar Paratyphi A (*S*. Para A) is ranked second as a causative agent of enteric fever, preceded only by *Salmonella enterica* serovar Typhi (*S*. Typhi). Enteric fever caused by *S*. Para A, or Paratyphoid fever was thought to be responsible for a comparatively smaller proportion of enteric fever cases [1]. However, since the 1980s both the incidence and relative frequency of Paratyphoid fever have risen in Nepal, Pakistan, and Thailand [2, 3]. Moreover, the populous nations of India and China have reported substantial numbers of *S*. Para A cases [4, 5]. Non-endemic countries like the United States report an increasing trend of Paratyphoid fever especially, amongst travelers from South Asia [6].

*S*. Para A resides in the human gut and its clinical manifestations are indistinguishable from Typhoid fever. Its genome is like that of *S*. Typhi, with the additional accumulation of 173 to 210 pseudo genes among its protein coding sequences [7]. Until recently, *S*. Para A was mostly observed to have a distinct prevalence rate and drug resistance pattern from that of *S*. Typhi [8]. For example, multidrug resistant (MDR) *S*. Typhi strains, are commonly isolated from many enteric fever endemic countries, while MDR *S.* Para A strains are either not yet reported from many enteric fever endemic countries like Bangladesh and Nepal [9, 10] or infrequently reported from some countries like Pakistan and India [11, 12]. Likewise, while ceftriaxone resistant *S*. Typhi strains have been commonly reported from Pakistan, this resistance has not been reported in *S.* Para A from Pakistan. In fact, our extensive literature search has revealed only one case report of ceftriaxone resistant *S.* Para A, published from United Kingdom in 2020. Ceftriaxone resistant *S.* Para A in that case was causing infective colitis in a traveler returning to England from Bangladesh [13].

Here, we report the first ceftriaxone resistant* S*. Para A, isolated from the blood culture of a clinically suspected case of paratyphoid fever or enteric fever.

## Case presentation

On 1^st^ June 2021, a set of blood culture bottle (BACT/ALERT® Culture Media, bioMérieux) was received at the Clinical Microbiology Laboratory of Aga Khan University Hospital (AKUH) Karachi, Pakistan. The patient was a 29-year-old female, resident of Karachi with no known comorbidity. On 3^rd^ day of fever, headache, and shivering, she consulted her physician who requested blood culture. After positive blood culture result, she was prescribed oral Azithromycin for 10 days, her symptoms subsided with medications.

*S*. Para A (isolate S7) was identified using API® 20E (Analytical profile index-BioMérieux) and Salmonella poly O and factor 2 antisera (BD-difcoTM). Antimicrobial drug susceptibility testing was performed by Kirby-Bauer disk diffusion and VITEK 2® COMPACT SYSTEM (BioMérieux). Susceptibility results against ampicillin, ceftriaxone, cefixime, chloramphenicol, trimethoprim-sulfamethoxazole, ciprofloxacin, ofloxacin, azithromycin, imipenem, ertapenem and meropenem were interpreted using recent CLSI performance standards for Antimicrobial Susceptibility Testing (M100-Ed32).

Two additional *S*. Para A clinical isolates (S1 and S4) were randomly picked from the same batch of positive blood cultures and underwent same identification protocol like *S*. Para A (S7). Similarly, S1 and S4 isolates were tested against same antibiotics as *S*. Para A(S7) using similar susceptibility testing methods.

Whole genome sequencing (WGS) was performed on DNA extracted from *S*. Para A isolates S1, S4 and S7. Library preparation was performed using the Nextera XT DNA kit. Sequencing was performed on the Illumina MiniSeq system. FASTQ files of the samples were checked for quality using Fastqc v0.11.9 (https://github.com/s-andrews/FastQC) and were trimmed and assembled using Shovill v0.9.0 (https://github.com/tseemann/shovill). The assembly quality was checked using QUAST [14] using the reference genome *S*. Para A (NC_006511) [7]. Variant calling was performed using Freebayes v1.3.5 [15] and Snippy v4.6.0 (https://github.com/tseemann/snippy) and the variants were annotated using snpEFF v4.5.0 [16]. The AMR profiling was performed using rgi v5.1.1 [17] and staramr v0.7.2 [18] using CARD, Megares, AMRfinder databases.

**Fig. 1:**
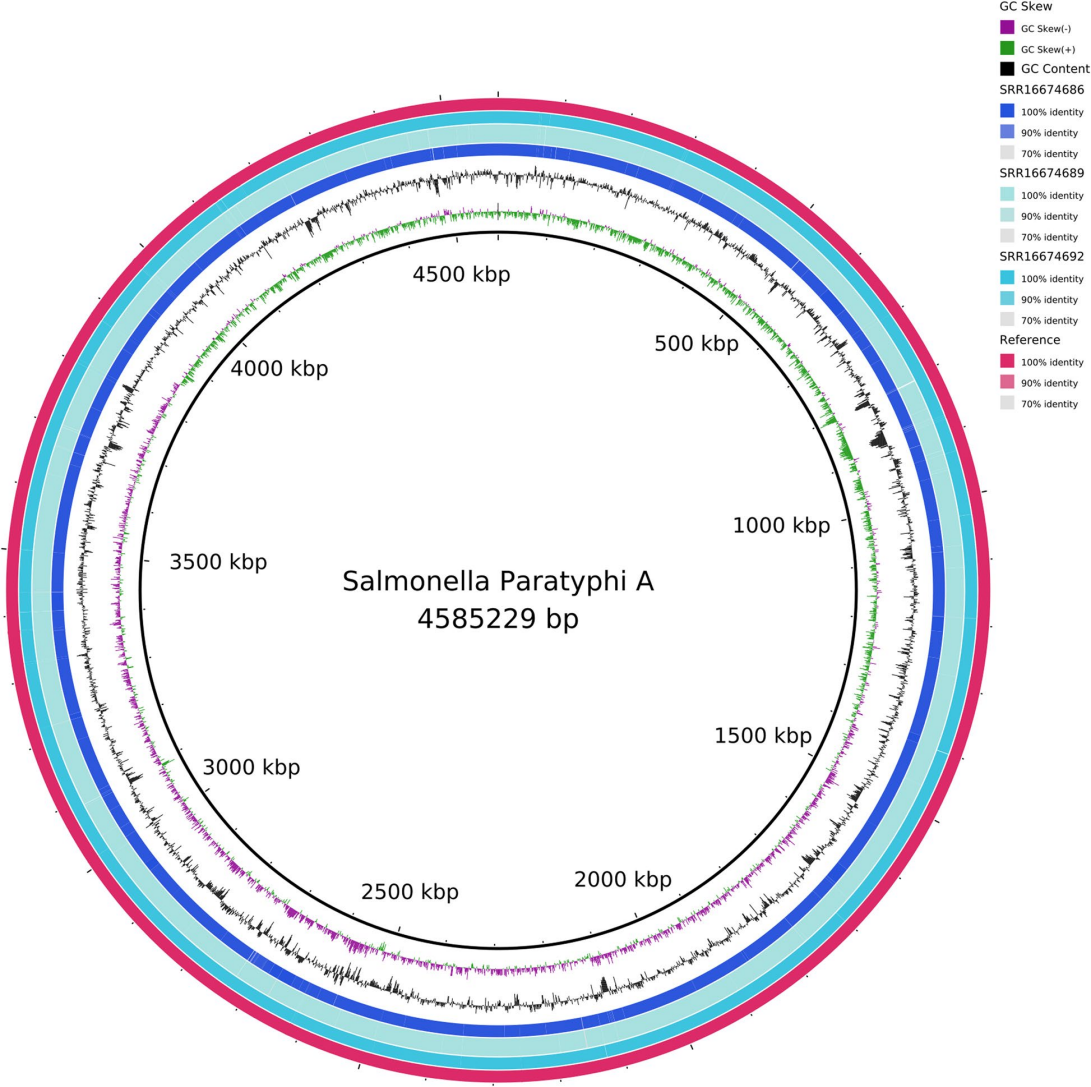
BRIG plot of *S*. Para A strains. The coverage map of three *S*. Para A sequences is shown as compared with the reference *S*. Para A strain (SRR7209524). Additional GC content and GC Skew features are displayed. The tool used to draw figure is BRIG tool (https://brig.sourceforge.net/)

*S*. Para A sequences (S1, S4 and S7) were submitted to NCBI SRA with accession numbers SRR16674692, SRR16674689 and SRR16674686, respectively. Genome sequences were compared with the *S*. Para A reference sequence accession number (NC_006511) [7], a sequence reported from a travel associated infective colitis caused by ESBL producing ceftriaxone resistant *S*. Para A, from stool sample of a traveler returning to England from Bangladesh in 2017 [13] and a *S*. Para A sequence from Bangladesh (SRR7209524), Fig. 1.

Resistance genes were determined using ResFinder v4.0 software [19]. PlasmidFinder v2.1 [20] was used to detect the types of plasmids. The gene cassettes were identified using the prokka tool [21].

**Fig. 2:**
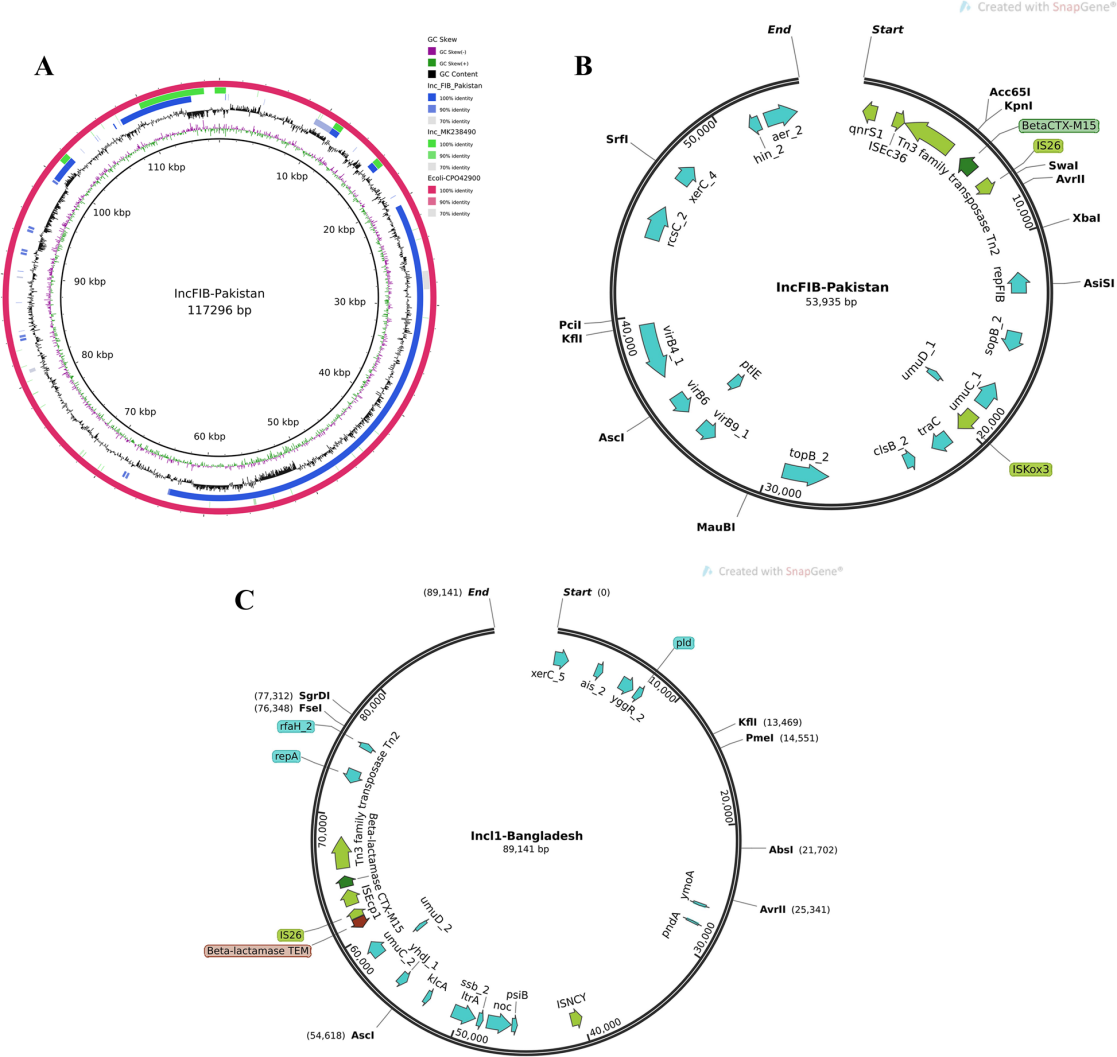
XDR plasmid in *S*. Para A isolate. **A** Pairwise alignment of S7 IncFIB(K) plasmid (blue ring) is shown against the *S*. Para A strain from Bangladesh with Inc-MK238490 (green ring) and E.coli plasmid CPO42900 (reference sequences, outer red ring). GC-skew is shown in inner ring, black line. The circular alignment is produced and visualized using BRIG tool. **B** expanded region (genes in red) indicating presence of beta*CTX-M-1* associated with transposase *Tn2* and *Tn3*. **C** Bangladesh strain plasmid Inc-MK238490 with beta*CTX-M-1* cassette with *Tn3* family genes. The plasmids are constructed using SnapGene tool (www.snapgene.com)

BLAST [22] pairwise alignment was performed to compare relatedness of plasmids between S7 IncFIB (Pakistan) and the Inc-MK238490 (Bangladesh) [13] (Fig. 2A). The plot was visualized using BRIG tool [23]. The Fig. 2B and C displayed with SnapGene (www.snapgene.com).

**Fig. 3:**
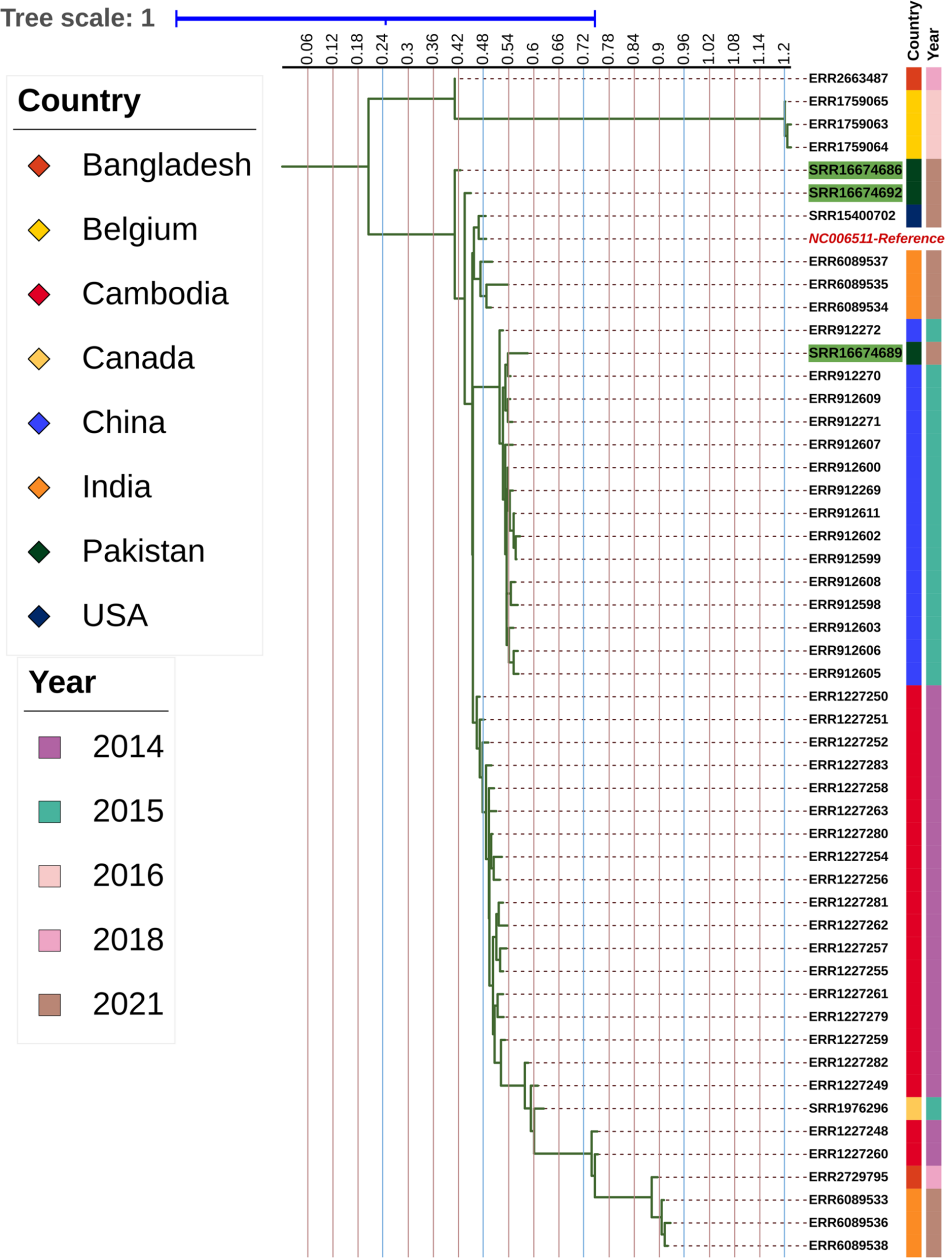
Relatedness of the three *S*. Para A strains with global isolates. S1 = SRR16674692, S4 = SRR16674689 and S7 = SRR16674686, with strains from countries of different regions: Cambodia, Canada, China, India, Bangladesh, Belgium, and USA. The strains were reported from 2014 to 2021

A phylogenetic tree was made using the variants generated using Snippy for sequences from S1, S4 and S7 along with the other available *S*. Para A sequences of strains reported from between 2014 and 2021 from countries of different regions: Cambodia [24], Canada [25], China [26], Bangladesh [27], Belgium (BioProject: PRJEB18573) and USA [7]. The tree was generated using Iqtree with (http://www.cibiv.at/software/iqtree) using substitution model GTR algorithm (Fig. 3). The tree was visualized using an interactive tree visualization tool itol [28].

Identification and assignment of species type for *S*. Para A (S1, S4, and S7) isolates were determined using Multi Locus Sequencing Typing MLST v2.19.0 [29].

## Results

Antimicrobial susceptibility testing results of S1, S4 and S7 isolates are shown in Table 1.

Ceftriaxone MIC for S7 was >  = 64 µg/ml and for SI and S4, it was <  = 1 µg/ml. All three isolates (S1, S4 and S7) were susceptible to imipenem, ertapenem, meropenem and azithromycin.

We found consistent sequence coverage across the whole genome of all three isolates (S1, S4 and S7) (Fig. 2A).

S1 had the fluoroquinolone resistance conferring mutation S83Y in the *gyrA* gene whilst S4 and S7 had *gyr*A S83F. S7 isolate was found to have *bla*_*CTX-M-15*_ gene associated with Ceftriaxone resistance and the *qnrS1* gene (Table 1). S7 isolate had two plasmids, IncX4 and IncFIB. *bla*_*CTX-M-15*_ and *qnrS1* genes were found on IncFIB, (Fig. 2B).

**Table 1:** S. Para A isolates, phenotypic and genotypic profiles

		**Phenotypic Profile**							**Genotypic resistance profile**			
Isolate #	Organism	Ceftriaxone	Cefixime	Ampicillin	trimethoprim-sulfamethoxazole	Chloramphenicol	Ciprofloxacin	Ofloxacin	Ceftriaxone	Ampicillin	Chloramphenicol	Ciprofloxacin/Ofloxacin
S1	*S.* Paratyphi A	S	S	S	S	S	S	I	-	-	-	gyrA (p.S83Y)
S4	*S.* Paratyphi A	S	S	S	S	S	S	I	-	-	-	gyrA (p.S83F)
S7	*S.* Paratyphi A	R	R	R	S	S	R	R	bla_CTX-M-15_	bla_TEM-1B_, bla_CTX-M-15_	catA1	qnrS1, gyrA (p.S83F)

We compared the IncFIB plasmid of S7 with the Inc-MK238490 plasmid of a MDR *S*. Para A isolate from Bangladesh [13] (Fig. 2C). Overall, there was less than 10% sequence similarity found between the plasmids from Pakistan (IncFIB) and Bangladesh (Inc-MK238490) strains. The Bangladeshi Inc-MK238490 plasmid had the *bla*_*CTX-M-15*_ and *bla*_*TEM-1B*_* genes*, the latter associated with Ampicillin resistance.

The *bla*_*CTX-M-15*_ gene in both the Pakistan and Bangladesh strains were associated with a transposon. The gene cassette *ISEcp9 –bla*_*CTX-M-15*_*-hp-tnpA* was present in the Bangladesh strain [13]. In S7 we found the *bla*_CTX-M-15_ gene to be associated with *ISEc36*-*tnp*3 (Fig. 2B and C).

Phylogenetic analysis of the Pakistani *S*. Para A isolates revealed that the S1 and S7 isolates were closely related to an isolate from Bangladesh identified in 2017 [13] (Fig. 3). Whilst the S4 isolate was related to an isolate identified from a 2010 community outbreak of paratyphoid fever in China [30]. We performed MLST analysis for the isolates which revealed that S1 and S7 belonged to ST129 while S4 belonged to ST85.

## Discussion

To our knowledge this is the first report of extended-spectrum-β-lactamase (ESBL) positive *S*. Para A (S7) from a Paratyphoid fever case. This S7 isolate shows antimicrobial resistance determinants identified by both phenotypic and genotypic methods. The S7 isolate was also genomically aligned with *S*. Para A sequence from Bangladesh (SRR7209524) which also harboured *bla*_CTX-M-15_ containing plasmid conferring resistance to cephalosporin [13]. The overall *blaCTX-M-15* containing plasmids similarity among *S*. Para A strains from Pakistan and Bangladesh was found low (< 10%). They were also different incompatibility groups, IncFIB in this study and Inc-MK238490 from the Bangladesh strain [13]. However, strains from both Pakistan and Bangladesh included *bla*_*CTX-M-15*_ gene with a transposon associated with it. The gene cassette *ISEcp9 –*_CTX-M-15_-hp-tnpA has been reported to be responsible for transmission of the ESBL resistance in the Bangladesh strain [13]. In our strains however we found the *bla*_*CTX-M-15*_ gene to be associated with *ISEc36-tnp3*. This data supports the role of transposons in the transfer of AMR genes between plasmids of different strains.

It is of interest that both the S7 isolate here, and the *S*. Para A isolate from Bangladesh (SRR70209524) belonged to the ST129 clade. It would be necessary to conduct experimental studies to investigate whether this was a coincidence or that ST129 may be more readily available to accept plasmids from ESBLs harboring *bla*_CTX-M-15_.

*S*. Para A has an established dissimilarity in the prevalence rate and drug resistance pattern from its peer organism *S*. Typhi [8]. In Pakistan, MDR *S*. Para A strains have shown a sustained resistance rate of 2.2% since 2009, while a high percentage of fluoroquinolone resistant *S*. Para A strains has been reported from this country [12]. The S7 isolate has shown phenotypic and genotypic resistant to ampicillin, fluoroquinolone, and ceftriaxone. Though this isolate was also carrying *catA1* gene, however, phenotypically it appeared susceptible to chloramphenicol. Ceftriaxone resistance was conferred by plasmids IncFIB. This plasmid type has been shown to carry resistance genes in Enterobacteriaceae [31]. Resistance to fluoroquinolone may be conferred by either the presence of *qnr* genes or mutations in the *qrdr* (quinolone resistance determining region of *gyrA* genes. As such, the intermediate resistance to fluoroquinolone observed in strains S1 and S4 could be due to mutations in *gyrA* S83F. Mutations in the *qrdr* have been defined as codons 67–106 in *gyr*A. These genes encode for drug target enzymes, DNA gyrase and DNA topoisomerase IV, and therefore mutations in these genes are associated with fluoroquinolone resistance [32].

Our study highlights the emergence of rising drug resistance in *S*. Para A. Increased drug resistance in *S*. Typhi and *S*. Para A is observed globally and associated with spread through travelers [33]. Expanding ESBL resistance in *S*. Para A and other Typhoidal *Salmonella* will increase the pressure in empirical treatment of Typhoidal salmonellae with treatment providers resorting to expensive injectable such as, meropenem and the oral macrolide, Azithromycin. Overuse of azithromycin is already raising a threat for surge in its resistance through the COVID-19 pandemic [34]. Due to potential to spread by fecal oral route, increasing drug resistance in *S.* Para A is a public health concern. Given that the current Typhoid conjugate vaccine does not provide defense against paratyphoid fever, and the fact that *S*. Para A reveals a different AMR profile from its peer *S*. Typhi, enteric fever disease dynamics and usefulness of empirical therapy are likely to change soon. Therefore, gaining further knowledge about *S*. Para A is essential. Furthermore, there is a need to introduce *S*. Para A vaccine for the at-risk population.

## Conclusion

This case report indicates paratyphoid fever (enteric fever) causing potential of plasmid-mediated ceftriaxone resistant strain of *S*. Para A. The presence and emergence of ESBL *S*. Para A in Pakistan indicates the importance of epidemiological surveillance to track and monitor antimicrobial resistance in this pathogen. The emergence of this resistant *S.* Para A indicates additional threat due to drug resistant Salmonella species and highlights the importance of public health preventive measures and enhanced vaccinations to prevent further spread.

## Data Availability

Data sharing is not applicable to this article as no datasets were generated or analyzed during the current study.

## Abbreviations

*S*. Para A: *Salmonella enterica* Serovar Paratyphi A
*S*. Typhi: *Salmonella enterica* Serovar Typhi
LMIC: Low- and middle-income countries
AMR: Antimicrobial resistance
MDR: Multidrug resistant
ESBL: Extended-spectrum-β- lactamase
MLST: Multi-locus sequence typing
AKUH: Aga Khan University Hospital
qrdr: Quinolone resistance determining region
HSP: Health Security Partners
