# Correction: Ceftriaxone resistant Salmonella enterica serovar Paratyphi A identified in a case of enteric fever: first case report from Pakistan

**DOI:** 10.1186/s12879-023-08321-w

**Published:** 2023-05-23

**Authors:** Seema Irfan, Zahra Hasan, Farah N Qamar, Najia Ghanchi, Javaria Ashraf, Akbar Kanji, Safina Abdul Razzak, David Greig, Satheesh Nair, Rumina Hasan

**Affiliations:** 1 Department of Pathology and Laboratory Medicine, The Aga Khan University, Stadium Road, P.O Box3500, Karachi, 74800 Pakistan; 2 Department of Pathology and Laboratory Medicine, The Aga Khan University, Karachi, Pakistan; 3 Department of Pediatrics and Child Health, The Aga Khan University, Karachi, Pakistan; 4 Gastrointestinal Bacteria Reference Unit, UK Health Security Agency, London, UK; 5 GASTROINTESTINAL PATHOGENS UNIT Gastrointestinal Bacteria Reference Unit National Infection Service, Public Health England, London, UK; 6 Faculty of Public Health and Policy, London School of Hygiene and Tropical Medicine, London, UK

**BMC Infectious Diseases (2023) 23:267**

10.1186/s12879-023-08152-9

The original publication of this article contained an incomplete authors name. The incorrect and correct information is listed in this correction article, the original article has been updated.

Incorrect

Safina Razzak and Farah Qamar

Correct

Safina Abdul Razzak and Farah N Qamar

